# Intra-Genomic Ribosomal RNA Polymorphism and Morphological Variation in *Elphidium macellum* Suggests Inter-Specific Hybridization in Foraminifera

**DOI:** 10.1371/journal.pone.0032373

**Published:** 2012-02-29

**Authors:** Loïc Pillet, Delia Fontaine, Jan Pawlowski

**Affiliations:** Department of Genetics and Evolution, University of Geneva, Geneva, Switzerland; Université Paris Sud, France

## Abstract

*Elphidium macellum* is a benthic foraminifer commonly found in the Patagonian fjords. To test whether its highly variable morphotypes are ecophenotypes or different genotypes, we analysed 70 sequences of the SSU rRNA gene from 25 specimens. Unexpectedly, we identified 11 distinct ribotypes, with up to 5 ribotypes co-occurring within the same specimen. The ribotypes differ by varying blocks of sequence located at the end of stem-loop motifs in the three expansion segments specific to foraminifera. These changes, distinct from typical SNPs and indels, directly affect the structure of the expansion segments. Their mosaic distribution suggests that ribotypes originated by recombination of two or more clusters of ribosomal genes. We propose that this expansion segment polymorphism (ESP) could originate from hybridization of morphologically different populations of Patagonian *Elphidium*. We speculate that the complex geological history of Patagonia enhanced divergence of coastal foraminiferal species and contributed to increasing genetic and morphological variation.

## Introduction

Member of the family Elphidiidae [1], *Elphidium macellum* is a common species of benthic foraminifera that occurs in coastal marine environment. It is frequent in temperate and sub-tropical low tidal and shallow subtidal ecosystems [2], especially in the Southern hemisphere where it sometimes represents a major component of the assemblage of foraminifera [2]–[4].

An interesting feature of Elphidiidae, is the high morphological variability observed between individuals of the same species and often within the same population [5]–[7]. These morphological variations are especially well documented in the case of *E. macellum* from Patagonia [8], [9]. In most of the previous studies dealing with this high level of morphological variability, the authors considered those changes as induced by environmental factors and they suggested that several described species could in fact be the ecophenotypes of the same species. Different authors tried to quantify the impact of environment on the morphology of the foraminiferan test [6], [10] and in parallel many studies focused on clarifying the taxonomic confusion introduced by different interpretations of morphological variants [10], [11]. Today, the taxonomic classification of elphidiids remains unclear and furthermore nothing is known about the molecular aspect of this intra-specific variability.

The initial aim of the present study was to establish the phylogenetic position of *E. macellum* among elphidiids and to test whether its high morphological variations were indicative of cryptic speciation or whether they were induced by environmental factors. To achieve this goal we sequenced a fragment of the SSU rRNA gene that is commonly used in molecular systematics of foraminifera [12]. Surprisingly, the results of the preliminary analyses not only confirmed a high level of variability at the intra-specific level, but also revealed the occurrence of a strong polymorphism within single individuals of *E. macellum*. Furthermore, this ribosomal polymorphism shows a very peculiar patterns consisting of nucleotides blocks that seems to be interchangeable between them and located in homogenous loci along the SSU rDNA sequence.

To further elucidate the origin of this polymorphism, 70 sequences of the partial SSU rRNA gene were obtained from 25 specimens of *E. macellum* collected in seven different localities in the Patagonian fjords. They were analysed to characterize the intra-specific and intra-genomic variability observed in this species. The predicted secondary structure of these partial SSU rRNA sequences was then modelled to localize the position of these variations and in order to understand the mechanism leading to such high ribosomal haplotypical diversity. To examine the position of *E. macellum* among its relative species, we established an updated phylogeny of the genus *Elphidium*. Considering the exceptional intra-genomic variability and the extraordinary ‘fragmented’ design of the SSU rDNA described above, the present study strongly suggests the occurrence of recombination within different sets of the SSU rRNA gene of *E. macellum*. Among various explanations proposed for the presence of different ribosomal genes within the same genome, the most parsimonious in our case seems the one involving inter-specific hybridization events between closely related species.

## Results

### Morphological variability in *E. macellum*

**Figure 1:**
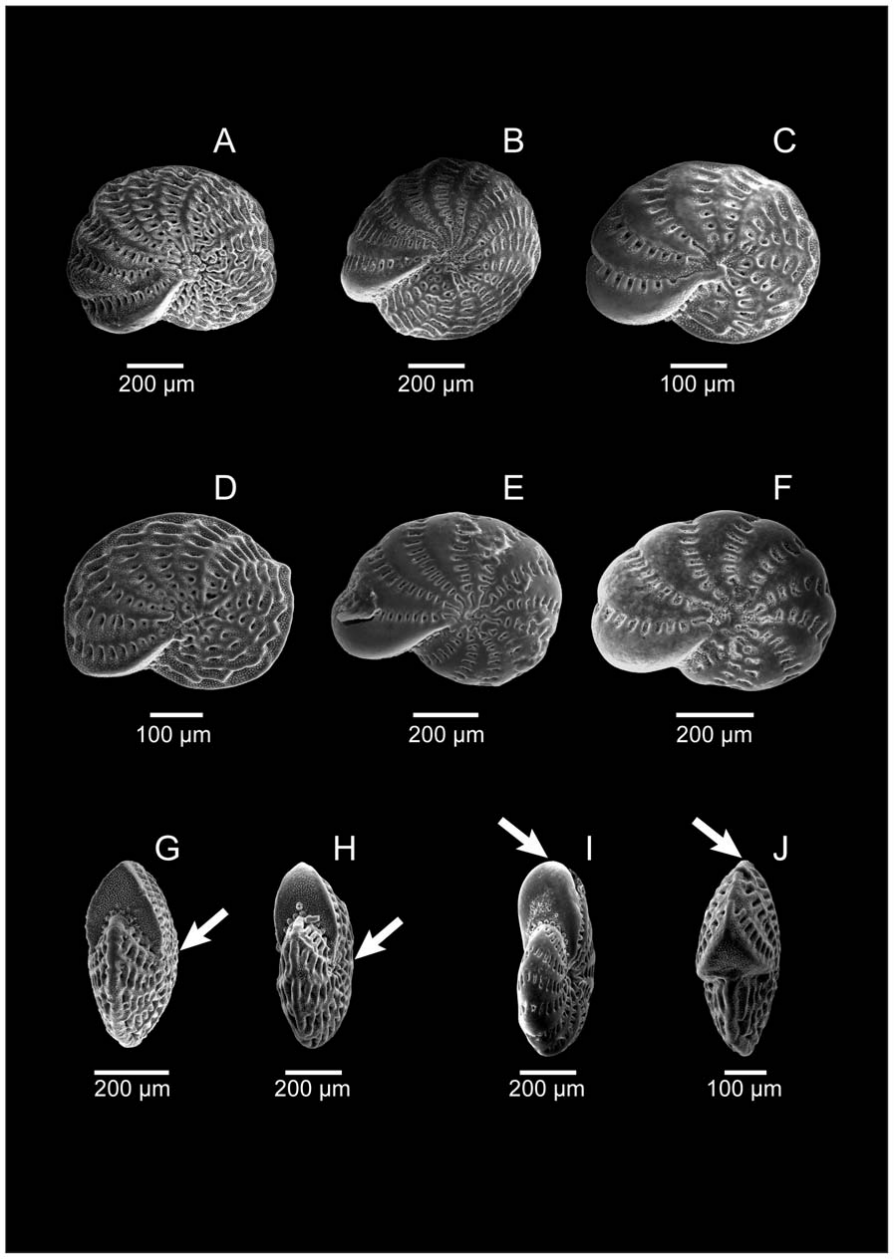
Scanning Electron Microscopy (SEM) pictures of various specimens of *Elphidium macellum*. Different individuals are shown on lateral (A–F) or on peripheral (G–J) view. For details about the terminology used to describe the important morphological characters, see Figure S1.

Many morphological characters were variable in different specimens of Patagonian *E. macellum* and some examples of this variability were illustrated in Figure 1 and Figure S1. The main changes were observed around the umbilical area; in the majority of collected individuals, this region was depressed (Figure 1H) although some specimens had a broadly inflated umbilical area, showing clear biconvex profiles (Figure 1G). The presence or absence of peripheral keel was also highly remarkable, as it influenced the shape of the last chamber and the apertural face. In some specimens the periphery was acute and thus the apertural face looked triangularly shaped (Figure 1J), whereas in others there was no keel and the periphery was rounded as well as the apertural face (Figure 1I). The number of chambers in the last whorl was also variable, usually around 13 (Figures 1C to E) but ranging from 10 (Figure 1F) to 17 (Figures 1A and B). The number of septal bridges per chamber varied between 7 (Figure 1C) and 12 (Figures 1A and B) and in certain specimens they were long, prominent and seemed to cover the whole surface of the test (Figures 1A and B) whereas in others they were less important (Figures 1E and F). Ornamentation of the test was also a very variable feature in *E. macellum*. Some specimens were strongly ornamented with granules covering the surface of the test excluding the septal bridges (Figures 1A to D) but including the apertural face (Figures 1G, H and J). In other individuals, this kind of ornamentation was restricted to the intercameral sutures (Figures 1E and F) and the rest of the test including the apertural face seemed very smooth (Figure 1I).

### SSU rDNA phylogeny of *Elphidium*

**Figure 2:**
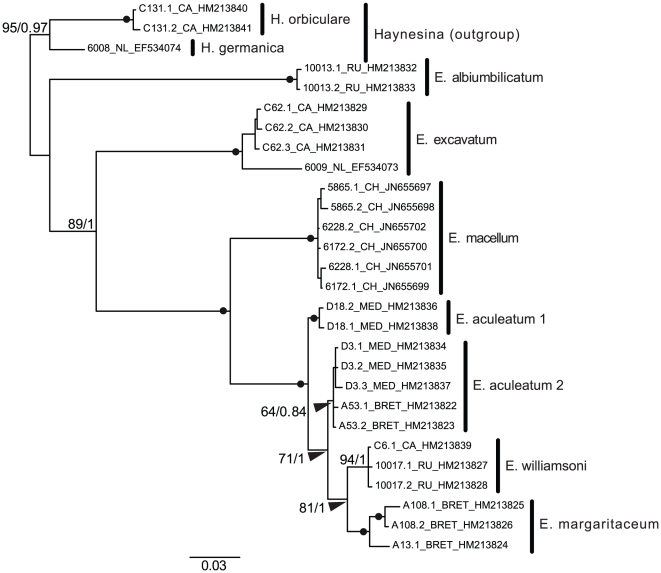
Elphidiids complete SSU rDNA phylogeny. Bayesian phylogeny implemented using the GTR+*Γ* model of evolution. RAxML bootstrap values and MrBayes posterior probabilities are shown at the nodes and solid circles indicate maximum node support (100/1.0). For each sequence, the DNA number is followed by the sampling location: CA (Halifax), RU (White Sea), NL (North Sea), CH (Patagonia), MED (Mediterranean Sea) and BRET (Brittany) and by the GenBank accession number.

Maximum likelihood and Bayesian phylogenetic analyses led to the same tree topology (Figure 2), highlighting eight different molecular clades corresponding in most of the cases to a morphologically determined species. The only ambiguous situation was found in the case of *E. aculeatum*, which was split into two different molecular clades: *E. aculeatum* 1 and *E. aculeatum* 2. All trees were rooted using as outgroup the *Haynesina* sister genus and the first elphidiid diverging species was always *E. albiumbilicatum*, followed by *E. excavatum* and *E. macellum*. The last diverging and sister clade to *E. macellum* was composed of four rapidly diverging species: *E. aculeatum* 1, *E. aculeatum* 2, *E. williamsoni* and *E. margaritaceum*. Most of the internal nodes were statistically well supported, with exception of *E. aculeatum* 2 (64/0.84), reflecting the complexity of this morphospecies, probably composed of different sub-species or cryptic species.

### Intra-genomic variability in *E. macellum*

Analysis of 70 partial sequences of the SSU rDNA obtained from 25 individuals of *E. macellum* showed particular variations in three hyper-variable regions specific to foraminifera (Figures S2, S3, S4). Interestingly, the changes in these regions not only occurred between different specimens of *E. macellum*, but were also observed between different clones obtained from the same individual. The most interesting feature of these three hyper-variable regions was that they were not composed of completely random nucleotide sequences, but each of them comprised one of two or three possible homogenous sequences. There were three possible sequences for the first region (Figure S2), two for the second region (Figure S3), and two for the third one (Figure S4). The size of these loci was variable; the first-one was 6, 8 or 10 bp long, the second one was 32 or 34 bp long and the third one was either 11 bp or 14 bp long, depending on which possible sequence was chosen.

**Figure 3:**
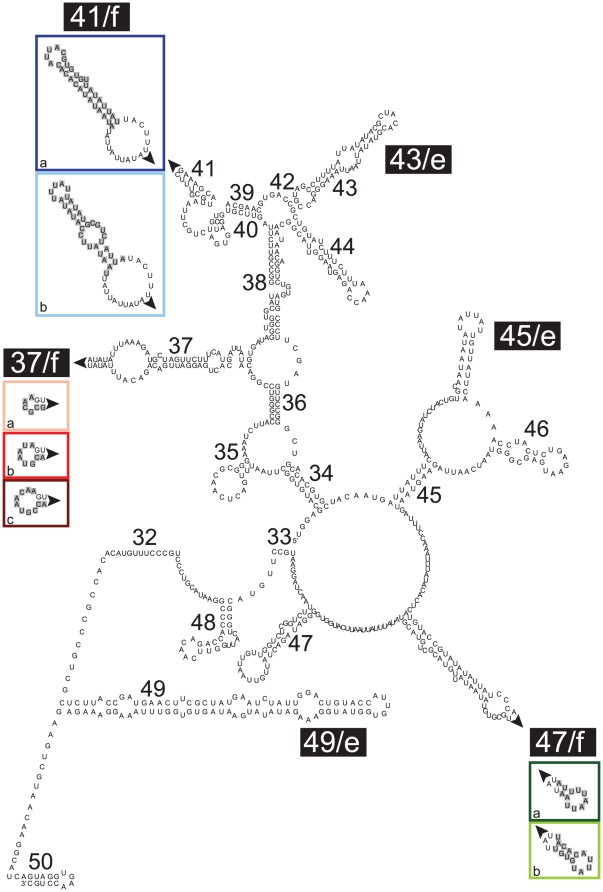
Predicted secondary structure of the partial SSU rRNA molecule of *Elphidium macellum*. The numbers correspond to the helices forming the region or located next to it. The six hyper-variable expansion segments found in eukaryotes (e) and those specific to foraminifera (f) are shown in black boxes. For each expansion segment specific to foraminifera, the ESPs are represented in separate colour boxes, where the variable positions of the rRNA molecule are shown in grey boxes. Different colours are used to characterize the different ESPs and the same colour code is used to show the ribotypical diversity in Figure 4.

To further characterize these different ribotypes, the secondary structure of the SSU rRNA of *E. macellum* was predicted and the three variable regions were located in this model (Figure 3). Single nucleotide polymorphism was not shown on that representation, as the present study focused on highly variable regions only. The nomenclature used to describe the predicted secondary structure was based on a previous study [13] and the variable regions were named according to numbers assigned to the helices forming the region or located next to it. The highly variable regions (1), (2) and (3) found in *E. macellum* were always located in the expansion segments at the end of a stem-loop motif, respectively named 37/f, 41/f and 47/f. The predicted secondary structure for the different type sequences found in each of these region were represented in separated boxes and named 37/f (a, b and c), 41/f (a and b) and 47/f (a and b). The variable positions were shown in grey boxes. Interestingly, in the first highly variable region (37/f), as only the size of the terminal loop changed, there was no dramatic architectural modification observed between the three different secondary structures. On the other hand, both regions 41/f and 47/f underwent important structural changes if the type sequence (b) was chosen. In the first case, a relatively large loop was added within the terminal part of the helix 41/f. In the second situation, a small loop was added inside the helix 47/f structure. These changes, distinct from typical Single Nucleotide Polymorphisms (SNPs) and insertion-deletions (indels), directly affect the structure of expansion segments and were called here ESPs (Expansion Segments Polymorphisms).

**Figure 4:**
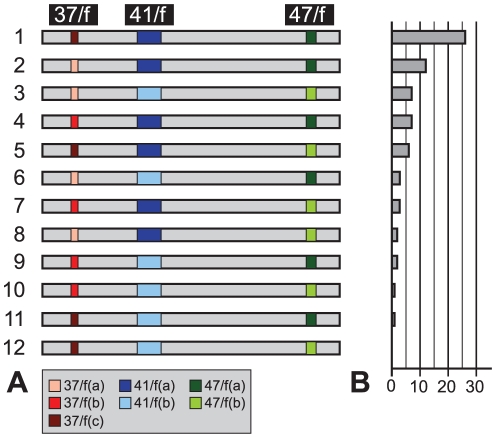
Ribotypical diversity in *Elphidium macellum*. The 12 different ribotypes composed of all different combinations of ESPs per locus are shown (A) and classified by abundance. The different colours used to characterize the different ESPs are the same used in Figure 3. In a total of 70 clones, the abundance of each ribotype is shown (B) and ranges from 26 for the ribotype number 1 to 0 for the ribotype number 12, which is not recovered in our analysis.

As three sequences were possible for the regions 37/f and two sequences were possible for regions 41/f and 47/f, there were theoretically 12 different possible combinations of ESPs and therefore 12 different ribotypes (Figure 4). The prevalence of one or more type of ESPs for each locus was tested by quantifying the occurrence of the alleles between and among the different specimens of *E. macellum*. These data were summarized in Table S1. In total and among all analyzed individuals, 11 different ribotypes were found (Figure 4). The only combination that was not retrieved corresponds to 37/f (c), 41/f (b) and 47/f (b). For each of the 25 specimens, between one and seven clones were sequenced and among these different individuals, the number of different ribotypes found within the same specimen was comprised between one and five (Table S1). The Spearman correlation coefficient highlighted a strong (ρ = 0.79) and highly significant (p<1e-6) correlation between the number of clones and the number of ribotypes. Considering the 37/f expansion segment and as type sequences (a), (b) and (c) were found respectively 24, 13 and 33 times, there was no significant prevalence of one type sequence among the others. However regarding both variable regions 41/f and 47/f, there was an apparent bias favouring the usage of the type sequence (a) in both situations. As showed by the predicted secondary structure, both regions 41/f and 47/f underwent more important changes if the type sequence (b) was chosen and this could explain the prevalence of the (a) sequence in those variable regions.

## Discussion

The nuclear ribosomal RNA genes are widely used for inferring protists phylogeny [14]–[16] and species identification [13], [17]. Arranged in tandem arrays and typically repeated several hundred to some thousand times, these genes encode for rRNA molecules that fold into secondary structures, which possess conserved core regions alternating with more variable sequence segments [18] that have been called expansion segments [19], V (variable) regions [20], [21] or D (divergent) regions [22]. Despite the presence of these variable regions, the rRNA gene sequence is globally well conserved across taxa and in most cases does not show polymorphic variation at the intra-specific and intra-genomic levels. Usually accepted as the mechanism leading to this sequence uniformity, the concerted evolution model [23] explains how the different copies of a multigene family can evolve ‘horizontally’ in a coordinate way through unequal crossing-over and gene conversion [24], [25]. One of the expectations resulting of the concerted evolution dogma [26] is that the DNA sequence is conserved between the different copies of repeated DNA within a single genome or individual. Therefore and according to that model of evolution, the occurrence of more than one ribosomal haplotype should be avoided or eventually only minor changes, such as SNP, could be tolerated.

However, during the past decades, a number of studies showed intra-specific and even intra-genomic variability of the rDNA in various organisms including bacteria [27], metazoans [28]–[30], fungi [31], [32], plants [33]–[35] and protists [36]–[38]. Foraminifers are notorious concerning the high level of molecular divergence found in their rDNA [39] and a previous study [40] already pointed out an unusual variability of the LSU rRNA gene copies within single individuals of the genus *Ammonia*, which is a close phylogenetic relative of the genus *Elphidium* [41]. However, these exceptional ribosomal polymorphisms, located in different coding (16S/18S, 28S, 5.8S and/or 5S) and/or non-coding (ITS) regions, were often considered as digressions to the concerted evolution model and supported by various explanations involving peculiar genomic structures and/or life styles.

The present study highlights an unusual level of intra-genomic variability that was never reported in previous studies. Although some of the less abundant ribotype patterns could be artificially created by PCR chimerization, it is important to notice that the number of ribotypes present in the different analyzed individuals could be underestimated, as suggested by the high Spearman correlation coefficient between the number of clones and the number of ribotypes (Table S1). The present study also describes a completely different type of rDNA polymorphism. More complex than SNPs or indels, the ESPs observed in *E. macellum* are generally uncompensated and affect directly the structure of the expansion segments where they occur. These ESPs share many characteristics with the intra-individual ribosomal polymorphisms described in preceding studies, but none of the explanations suggested previously completely match the observations presented here.

Previous studies already suggested that the expansion segments were particularly variable and that they are hosting the majority of mutations occurring in the rRNA genes of a freshwater crustacean [42] and human [43], [44]. Although this preferential localization is in agreement with the polymorphism observed in the present study, the type of mutations was mainly described as length variation due to short sequence repeats and explained by the ‘compensatory slippage’ mechanism [45], which is not the case in our analyses. Compensatory slippage could therefore not explain the type of mutations observed in *E. macellum*.

Another possible explanation of the ESP could be related to the particular organisation of ribosomal genes in *E. macellum*. One of the assumptions of the concerted evolution model is that the genes evolving in accordance with that theory have to be organized in tandem arrays and it has been shown in previous studies that the 5S ribosomal genes of several organisms, which repeats are dispersed among the genomes, are escaping the concerted evolution model. These studies performed on several filamentous fungi species [32], an amphibian [46], [47] and a loach fish [48] highlighted several ribotypes co-occurring in the same individual and suggested that in these exceptional cases where ribosomal genes were not organized in tandem arrays, the Birth-and-Death evolution model [25] was more appropriate to describe their dynamics. In that model, the genetic evolution is regulated by a balance between gene duplication, turnover and maintenance, which allows the occurrence of several haplotypes within the same genome. This explanation was also suggested in the case of the apicomplexan protist *Plasmodium* sp. [49] which bears two distinct copies of the 18S rRNA gene that are also dispersed throughout its genome. This peculiar organization has been postulated as a mechanism allowing the parasite to escape the concerted evolution and permit it to possess two ribotypes that are necessary for the different stages of its life. As today, nothing is known about the genomic structure of foraminifera, the organization of the ribosomal genes in this group remains mysterious. Ongoing genomic projects will bring clearer answers to that question but according to preliminary results the evidence for tandem array arrangement of the ribosomal genes has not yet been found in foraminifera and therefore we cannot exclude that the Birth-and-Death evolution model could explain the results of the present study. However, the number of ribotypes and the particular structure of the ESPs make this explanation quite doubtful.

Similarly, it is possible albeit quite unlikely that the ESP in foraminifera is related to the multinucleate nature of these organisms. Such explanation was brought up in the case of intra-individual ribosomal ITS polymorphism in the microsporidian parasite *Nosema bombi* [31] and in the green alga *Caulerpa racemosa* [50]. The authors suggested that the different ribotypes co-occurring within the same individual were in fact encoded in different nuclei. In such situation, the concerted evolution model would still be valid, but restricted to the nucleus compartment level. Indeed, the life-cycle of the genus *Elphidium* comprises the multinucleate stage in asexual generation, but it alternates more or less regularly with uninucleate stage in sexual generation [51], [52]. Furthermore this is true for the majority of foraminifera that has been examined [53].

In our opinion, the most plausible explanation of the ESP in *E. macellum* involves inter-specific hybridization. The fragmented pattern of the SSU rRNA gene with the presence of interchangeable blocks of sequences suggests that distinct populations or species of elphidiids with different ribotypes hybridized resulting in a ‘new *E. macellum* species’ bearing the different sets of the rRNA genes. As a result of successive reproductive cycles together with recombination, the SSU rDNA would have acquired its unusual ‘mosaic’ pattern. This hypothesis is supported by the fact that the ESP occurred only in expansion segments specific to foraminifera (37/f, 41/f, and 47/f). These highly variable and expressed regions are normally conserved at the intra-specific level [13], suggesting that the *E. macellum* ribosomal genes originate from the DNA of different species. This is also congruent with a previous study based on different species of oak trees [54] that highlighted the existence of different ribotypes among the same genomes and suggested that their presence was the result of inter-specific hybridization. The importance of hybridization is well documented in plants and metazoans [55], however few examples are available regarding hybridization in protists. Natural inter-specific hybridization has been suggested in dinoflagellates [56], [57] and recently highlighted in diatoms [58], [59] at the intra-specific level. If confirmed, the present study would be the first case of hybridization reported in foraminifera.

Interestingly, the hypothesis of inter-specific hybridization could explain the extraordinary diversity within *E. macellum*, not only in term of ribosomal gene structure, but also regarding its different morphologies. Morphology in foraminifera is a long-lasting question and has been the principal source of inspiration for many generations of “splitters” and “lumpers”, fuelling the debate on species identification. Trying to set the frontier between inter-specific morphotypes and intra-specific ecophenotypes, many studies focused on quantifying the morphological variation that was observed, especially in rotaliids [6], [7], [60], [61]. In the genus *Elphidium*, this variability has been especially well documented and discussed for *E. excavatum* [6]. The authors characterized five morphotypes of *E. excavatum* associated with different environmental variables, proposing to consider them as ecophenotypes rather than the distinctive subspecies [62].

We cannot exclude that the various morphotypes of *E. macellum* are ecophenotypes as we did not carry out a detailed survey of environmental conditions. However, the fact that we observed a high level of morphological variation among specimens collected in the same locality precludes their ecological significance. Moreover, the morphological characters that vary in *E. macellum*, such as the test outline and profile, the presence or absence of peripheral keel, the number of chambers and septal bridges, are commonly considered as species characteristics rather than as indicators of changing environmental conditions [63]. Therefore, it seems more accurate to consider the morphological variation in *E. macellum* as result of inter-specific hybridization rather than as a consequence of ecophenotypic changes.

We can speculate that the hybridization occurred between two *Elphidium* populations that resembled the two major morphotypes present in our samples: the first one (Figures 1A and B) characterized by a large number of chambers with the presence of many prominent septal bridges and ornamental granules on each chamber, and the second one (Figures 1E and F) showing less chambers in the last whorl with fewer and less prominent septal bridges and smoother test surface with less ornamentation. Although the morphological variants induced by hybridization events are generally thought to be intermediate to parents [64] it has been shown that sometimes the resulting morphologies resemble more to one of them [65], [66]. The result of hybridization could therefore be illustrated by the presence of two major morphotypes and their intermediate forms, which corresponds exactly to what is observed for *E. macellum*.

In fact, more than one hybridization event could be involved in the formation of modern Patagonian *E. macellum*. The complex geological history of the Southern Patagonia makes the episodes of inter-specific hybridization very likely. The presence of different forms of *E. macellum* has been reported in the fossil record of the region since the lower Miocene, older than 17 Ma [67]–[69]. Since successive glaciations [70] and oceanic transgressions [71] occurred within this period of time, it seems probable that the ancestral populations present in Southern South America were fragmented and re-associated several times, making the speciation and hybridizations events happening straightforwardly.

The hypothesis of inter-specific hybridization is the only one that could explain both the extraordinary morphological variability of *E. macellum* and its unusual intra-genomic rRNA polymorphism. However, this hypothesis needs further testing to evaluate the importance of hybridization in foraminifera. Noticeably, such molecular variability can be difficult to detect in phylogenetic analyses because the hyper-variable regions are usually discarded from the alignment and the number of sequenced rDNA copies per specimen is limited. Indeed in the present study *E. macellum* forms a compact clade supported by maximal bootstrap value and posterior probability (Figure 2). Our preliminary study involving sequencing of many clones for single specimens showed that the ESP can be found also in other foraminiferal species (A Weber, L Pillet and J Pawlowski, unpublished data), but it is unknown whether it is associated to morphological variations. The relations between genetic and morphological variations can be difficult to demonstrate. However, given a huge morphological diversity of foraminiferal species, we can predict that at least part of this diversity is due to the inter-specific hybridization, an evolutionary factor that curiously was totally ignored in foraminiferal research until now.

## Materials and Methods

### Sampling and DNA extraction

Living specimens of *Elphidium macellum* were collected along the Patagonian coast between latitudes 51°S and 55°S (Figure S5). Algae and surface sediment were taken at low tide, washed in seawater and sieved. The fractions comprised between 500 µm and 125 µm were collected and stored at temperatures close to those of sampling sites. Live specimens were picked within a week after sampling and cleaned with small paintbrushes in several changes of filtered seawater to avoid contaminant. Scanning Electron Microscopy (SEM) was used to record the morphology of 15 analyzed specimens prior to DNA extraction. Total DNA was extracted from single specimens using a guanidine buffer based protocol [12]. No specific permits were required for the described field studies, as the sampling locations were not privately owned or protected in any way. Furthermore these field studies did not involve endangered or protected species

### PCR amplifications

The complete foraminiferal SSU rRNA gene of three *E. macellum* individuals was amplified in different steps using foraminiferal specific primers [16].

For 25 specimens, a partial fragment of the SSU rDNA of approximately 800 bp was amplified. The primers s14F3 - s20R [12], [72] were used for the amplification and the primers s14F1- s20R [12] for the re-amplification (nested PCR). Hot Start PCR amplifications and re-amplifications were performed in a total volume of 50 µl with an amplification profile consisting of 35 cycles (25 cycles for the re-amplifications) of 30 s at 94°C, 30 s at 50°C, and 60 s at 72°C, followed by 5 min at 72°C for final extension.

### Cloning and Sequencing

After purification using High Pure PCR Purification Kit (Roche Diagnostics, Basel, Switzerland) or MinElute PCR Purification Kit (QIAGEN, Basel, Switzerland), positive PCR products were cloned. They were ligated in the Topo TA Cloning vector (InvitroGen, Basel, Switzerland) or in the pGEM-T vector system (Promega, Duebendorf, Switzerland), and cloned using chemically competent cells One Shot TOPO10 (InvitroGen). Sequencing was done with an ABI PRISM Big Dye Terminator Cycle Sequencing Kit using an ABI 3130XL DNA sequencer (Applied Biosystem, Rotkreuz, Switzerland), according to the manufacturer's instructions. By overlapping the different fragments, a complete nuclear SSU rRNA gene was obtained for three individuals. All sequences used in this study have been deposited in the GenBank database and their accession numbers are summarized in the Table S2.

### Phylogenetic Analyses

Six complete SSU rDNA sequences of *E. macellum* were obtained and compared to 19 sequences of five other morphospecies of genus *Elphidium* and three sequences of their sister genus *Haynesina*. This alignment was used to infer an updated phylogeny of the genus *Elphidium* and to determine the position of *E. macellum* among this phylogenetic tree. This dataset was first aligned using MAFFT v.6 [73], [74] and then improved manually using BioEdit Sequence Alignment Editor [75]. Then, the Perl script MrAIC 1.4.3 [76] in combination with PHYML v2.4.4 [77] was used to choose the best model of sequence evolution by the Akaike Information Criterion (AIC). Applying the obtained settings a Bayesian method and a Maximum Likelihood (ML) method [78] were used to infer phylogeny. With the MrBayes program [79], two independent analyses were performed at the same time with four simultaneous chains (one cold and three heated) ran for 10,000,000 generations, and sampled every 1,000 generations. After having discarded 2,000 of the initial trees as burn-in, the consensus tree with the corresponding posterior probabilities (PP) was calculated for each data set. The ML method was implemented with the RAxML-HPC v7.0.4 software [80] and the reliability of internal branches was assessed using the RAxML rapid bootstrap method with 100 replicates [81]. Most of all bioinformatic analyses were carried out on the freely available Bioportal (http://www.bioportal.uio.no).

### Secondary structure prediction

The secondary structure of the SSU rRNA of *E. macellum* was determined using the RNAfold server [82] available on the Vienna RNA web servers (http://rna.tbi.univie.ac.at). The energy parameters of the model were rescaled to a temperature of 8°C, which corresponds to the average sea temperature at sampling sites. It was then manually improved using as a template the previously published secondary structure of the foraminiferan *Micrometula hyalostriata* [13].

## Supporting Information

Figure S1 **Terminology of the important morphological characters of the test outline (A) and profile (B) of** ***Elphidium macellum*** **.** um: umbilical area; pk: peripheral keel; af: apertural face; sb: septal bridge; is: intercameral suture.
(EPS)

Figure S2 **First hyper-variable region (37/f) of the SSU rDNA.** The three different ribotypes (a, b and c) found in *E. macellum* are shown and compared to *E. aculeatum* sequences (outgroup).
(EPS)

Figure S3 **Second hyper-variable region (41/f) of the SSU rDNA.** The two different ribotypes (a and b) found in *E. macellum* are shown and compared to *E. aculeatum* sequences (outgroup).
(EPS)

Figure S4 **Third hyper-variable region (47/f) of the SSU rDNA.** The two different ribotypes (a and b) found in *E. macellum* are shown and compared to *E. aculeatum* sequences (outgroup).
(EPS)

Figure S5 **Sampling localities in Chile and Argentina.**
(EPS)

Table S1 **Ribotype occurrence among the analyzed specimens for loci 37/f, 41/f and 47/f.**
(PDF)

Table S2 **Accession numbers of DNA sequences used in this study.**
(PDF)
