# Supplementary material for: Intra-Genomic Ribosomal RNA Polymorphism and Morphological Variation in Elphidium macellum Suggests Inter-Specific Hybridization in Foraminifera
Source: PLoS One. 2012 Feb 29;7(2):e32373. doi: 10.1371/journal.pone.0032373 (PMC3290570; doi:10.1371/journal.pone.0032373)
Supplement: Table S1 — Ribotype occurrence among the analyzed specimens for loci 37/f, 41/f and 47/f. (PDF) [file pone.0032373.s006.pdf]

Table S1. Ribotype occurrence among the analyzed specimens for loci 37/f, 41/f and 47/f.

DNA number	Number of clones	Number of ribotypes	37/f			41/f		47/f	
			**a**	**b**	**c**	**a**	**b**	**a**	**b**
14.662	2	2	1	1		2		1	1
18.666	2	2	1	1		1	1	1	1
2.649	2	1			2	2		2	
4.651	2	2	1	1		2		2	
5.652	1	1			1	1		1	
7.654	2	2			2	2		1	1
B6228	4	4	2	1	1	2	2	2	2
B6232	1	1			1	1		1	
B6236	2	2	1		1	1	1	1	1
C5865	5	1			5	5		5	
C5867	2	2	1		1	2		2	
G5861	3	2			3	2	1	3	
G5862	3	3	2		1	2	1	2	1
G5863	3	2	1		2	3		3	
L5749	1	1			1	1		1	
L5750	6	3	1		5	6		4	2
L5752	2	2	1	1		2		2	
L5808	2	2	1		1	2		1	1
L5809	2	2	1		1	2		2	
L5812	7	5	1	3	3	6	1	2	5
Ot6026	1	1		1		1		1	
Ot6172	6	4	4	2		2	4	3	3
Ot6174	1	1			1	1		1	
U6435	5	4	3	2		3	2	5	
U6436	3	3	2		1	2	1	2	1
**TOTAL**			**24**	**13**	**33**	**56**	**14**	**51**	**19**

Spearman correlation coefficient between number of clones and number of ribotypes:

$\rho = 0.7939$ with $p < 0.000001$
