# Supplementary material for: Intra-Genomic Ribosomal RNA Polymorphism and Morphological Variation in Elphidium macellum Suggests Inter-Specific Hybridization in Foraminifera
Source: PLoS One. 2012 Feb 29;7(2):e32373. doi: 10.1371/journal.pone.0032373 (PMC3290570; doi:10.1371/journal.pone.0032373)
Supplement: Table S2 — Accession numbers of DNA sequences used in this study. (PDF) [file pone.0032373.s007.pdf]

Table S2. Accession numbers of DNA sequences used in this study.

Organism / Gene	Morphospecies / DNA number	Accession number (s)
Foraminifers / Complete 18S rDNA	*H. orbiculare* / C131.1	HM213840
	H. orbiculare / C131.2	HM213841
	H. germanica / 6008	EF534074
	E. albiumbilicatum / 10013.1	HM213832
	E. albiumbilicatum / 10013.2	HM213833
	E. excavatum / C62.1	HM213829
	E. excavatum / C62.2	HM213830
	E. excavatum / C62.3	HM213831
	E. excavatum / 6009	EF534073
	E. macellum / 5865.1	JN655697
	E. macellum / 5865.2	JN655698
	E. macellum / 6172.1	JN655699
	E. macellum / 6172.2	JN655700
	E. macellum / 6228.1	JN655701
	E. macellum / 6228.2	JN655702
	E. williamsoni / C6.1	HM213839
	E. williamsoni / 10017.1	HM213827
	E. williamsoni / 10017.2	HM213828
	E. aculeatum / D3.1	HM213834
	E. aculeatum / D3.2	HM213835
	E. aculeatum / D3.3	HM213837
	E. aculeatum / D18.1	HM213838
	E. aculeatum / D18.2	HM213836
	E. aculeatum / A53.1	HM213822
	E. aculeatum / A53.2	HM213823
	E. margaritaceum / A13.1	HM213824
	E. margaritaceum / A108.1	HM213825
	E. margaritaceum / A108.2	HM213826
Foraminifers / Partial 18S rDNA	*E. macellum* / 14.662A	JN655703
	E. macellum / 14.662B	JN655704
	E. macellum / 18.666A	JN655705
	E. macellum / 18.666B	JN655706
	E. macellum / 2.649A	JN655707
	E. macellum / 2.649B	JN655708
	E. macellum / 4.651A	JN655709
	E. macellum / 4.651B	JN655710
	E. macellum / 5.652	JN655711
	E. macellum / 7.654A	JN655712
	E. macellum / 7.654B	JN655713
	E. macellum / B6228A	JN655714
	E. macellum / B6228B	JN655715
	E. macellum / B6228c	JN655716
	E. macellum / B6228d	JN655717
	E. macellum / B6232A	JN655718
	E. macellum / B6236A	JN655719
	E. macellum / B6236B	JN655720
	E. macellum / C5865A	JN655721
	E. macellum / C5865B	JN655722
	E. macellum / C5865C	JN655723
	E. macellum / C5865D	JN655724
	E. macellum / C5865E	JN655725
	E. macellum / C5867A	JN655726
	E. macellum / C5867B	JN655727
	E. macellum / G5861A	JN655728
	E. macellum / G5861b	JN655729

E. macellum / G5861c	JN655730
E. macellum / G5862A	JN655731
E. macellum / G5862B	JN655732
E. macellum / G5862C	JN655733
E. macellum / G5863A	JN655734
E. macellum / G5863B	JN655735
E. macellum / G5863C	JN655736
E. macellum / L5749	JN655737
E. macellum / L5750A	JN655738
E. macellum / L5750B	JN655739
E. macellum / L5750C	JN655740
E. macellum / L5750D	JN655741
E. macellum / L5750E	JN655742
E. macellum / L5750F	JN655743
E. macellum / L5752A	JN655744
E. macellum / L5752B	JN655745
E. macellum / L5808A	JN655746
E. macellum / L5808B	JN655747
E. macellum / L5809A	JN655748
E. macellum / L5809B	JN655749
E. macellum / L5812A	JN655750
E. macellum / L5812B	JN655751
E. macellum / L5812C	JN655752
E. macellum / L5812D	JN655753
E. macellum / L5812E	JN655754
E. macellum / L5812F	JN655755
E. macellum / L5812G	JN655756
E. macellum / Ot6026	JN655757
E. macellum / Ot6172A	JN655758
E. macellum / Ot6172B	JN655759
E. macellum / Ot6172c	JN655760
E. macellum / Ot6172d	JN655761
E. macellum / Ot6172e	JN655762
E. macellum / Ot6172f	JN655763
E. macellum / Ot6174	JN655764
E. macellum / U6435a	JN655765
E. macellum / U6435b	JN655766
E. macellum / U6435c	JN655767
E. macellum / U6435e	JN655768
E. macellum / U6435f	JN655769
E. macellum / U6436a	JN655770
E. macellum / U6436b	JN655771
E. macellum / U6436c	JN655772
